# TME remodeling and clinical challenges of immune checkpoint blockade in nasopharyngeal carcinoma

**DOI:** 10.3389/fonc.2026.1850783

**Published:** 2026-05-28

**Authors:** Chan Ou, Lu Qi, Haoyue Lai, Juan Ye, Xin Tian, Zhongwen Li

**Affiliations:** 1 Department of Head and Neck Oncology, The Second Affiliated Hospital of Zunyi Medical University, Zunyi, China; 2 Zunyi Medical University, Zunyi, China

**Keywords:** drug resistance, Epstein-Barr virus, head and neck cancer, immune checkpoint blockade, immunotherapy, nasopharyngeal carcinoma, tumor microenvironment

## Abstract

Nasopharyngeal carcinoma (NPC) is an Epstein-Barr virus (EBV)-driven malignancy characterized by a profoundly immunosuppressive tumor microenvironment (TME) that severely limits the efficacy of immune checkpoint blockade (ICB). In the modern comprehensive treatment era, the 5−year overall survival rate for non−metastatic NPC has reached 80-90%, and the prognosis of newly diagnosed non−metastatic patients is substantially better than that of more prevalent cancers such as lung and colorectal cancer. Although ICB has improved survival in recurrent/metastatic NPC, clinical benefits are restricted by primary and acquired resistance, the lack of effective predictive biomarkers, and immune-related adverse events. In this review, we elaborate on the core mechanisms by which EBV orchestrates immune evasion by shaping immunosuppressive cell infiltration, T-cell dysfunction, metabolic disorders, and physical stromal barriers. We systematically summarize recent advances in ICB-mediated remodeling of the NPC TME, including the reversal of T-cell exhaustion, restoration of metabolic balance, and normalization of vascular and stromal compartments. We highlight the unique EBV-driven resistance programs, including antigen silencing, compensatory immune checkpoint expression, and exosomal miRNA-mediated remote immunosuppression. We also discuss major challenges in current biomarker development and the clinical management of immune-related toxicities. Finally, we propose future strategies focusing on EBV-targeted intervention, multi-omics-based predictive models, and mechanism-driven combination immunotherapy to overcome resistance and improve precision treatment. This review provides a comprehensive mechanistic and translational overview that may facilitate the rational design of novel immunotherapeutic approaches for EBV-associated NPC.

## 1 Introduction

Nasopharyngeal carcinoma (NPC) is an EBV-driven head and neck malignancy with highly imbalanced geographic distribution, especially prevalent in South China and Southeast Asia (1). For patients with non-metastatic disease at diagnosis, the 5-year overall survival rate has reached 80–90% in the modern comprehensive treatment era, which is substantially better than that of more prevalent cancers such as lung and colorectal cancer (2, 3). Despite standard chemoradiotherapy, approximately 30% of patients develop recurrence or distant metastasis, resulting in a dismal prognosis (4). The advent of ICB has brought pivotal therapeutic advances for recurrent/metastatic NPC (R/M NPC). PD-1 inhibitors combined with chemotherapy have been recommended as first-line therapy for R/M NPC in the National Comprehensive Cancer Network (NCCN) guidelines (5). However, the clinical benefit of ICB is limited by substantial interpatient heterogeneity, frequent primary and acquired resistance, the lack of robust predictive biomarkers, and irAEs (6, 7). Mechanistically, EBV systematically establishes a highly immunosuppressive, metabolically dysregulated, and stroma-dense TME via latent proteins and non-coding RNAs. This not only fosters tumor immune evasion but also represents a central bottleneck for ICB efficacy (7). Therefore, clarifying the immune-remodeling mechanisms of the EBV-shaped TME and dissecting the multifaceted effects and resistance drivers of ICB are critical for advancing precision immunotherapy in NPC.

This review integrates cutting-edge advances in single-cell multi-omics and spatial transcriptomics. From the cellular, metabolic, and structural dimensions of ICB-mediated TME remodeling, we deeply analyze its molecular mechanisms and clinical challenges, and highlight emerging personalized combinatorial strategies based on TME spatiotemporal heterogeneity, to offer theoretical and translational perspectives for optimizing NPC immunotherapy.

## 2 EBV-driven immunosuppressive tumor microenvironment

The immunosuppressive microenvironment of NPC is a structured and dynamically evolving complex system whose formation and maintenance are primarily driven by persistent EBV infection. Virally encoded latent proteins (e.g., LMP1, LMP2, EBNA1) and non-coding RNAs (e.g., EBERs) reshape the local immune landscape through multi-dimensional mechanisms, forming a robust barrier for (8).

### 2.1 Driving immune reprogramming

**Figure 1:**
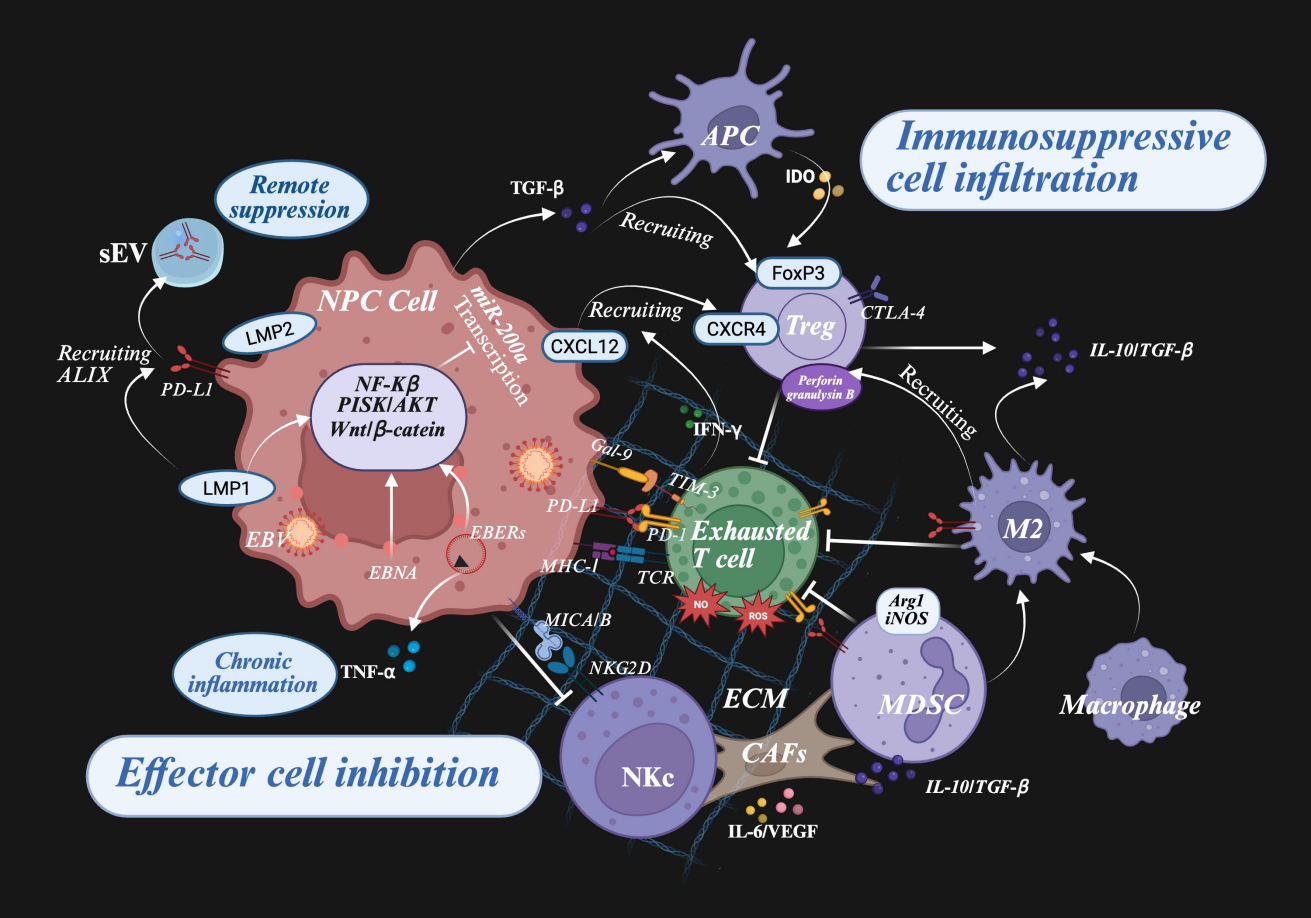
Mechanisms of the EBV-driven immunosuppressive microenvironment in NPC. EBV-infected NPC cells upregulate PD-L1 via LMP1 and downregulate the NK-activating ligand MICB through miRNAs. Tumor cells secrete chemokines (CXCL12, CCL2) to recruit TAMs, MDSCs, and Tregs, which produce IL-10, TGF-β, and metabolic enzymes (ARG1/iNOS) to establish immunosuppression. CD8^+^ T cells, NK cells, and DCs become dysfunctional with high PD-1, decreased cytokine secretion, and impaired antigen presentation. Blunt arrows: inhibition; solid arrows: induction or direction. Created with BioRender.com.

EBV-positive NPC cells directly initiate an immunosuppressive microenvironment by expressing a series of latent membrane proteins (**Figure 1**) (9). Latent membrane protein 1 (LMP1) upregulates PD-L1 expression in NPC cells and recruits ALIX to form a complex with PD-L1, promoting PD-L1 secretion via small extracellular vesicles (sEVs) to achieve remote immunosuppression (10). Meanwhile, LMP1 stimulates the secretion of IL-18 and IP-10 by activating multiple signaling pathways, including NF-κB, PI3K-AKT, and ERK-MAPK, thereby inducing IFN-γ production in activated T cells and NK cells. The JAK-STAT pathway activated by IFN-γ further induces PD-L1 expression in NPC cells, indicating a synergistic effect between LMP1 and IFN-γ in PD-L1 regulation, which has been confirmed in NPC models (11).

Epstein-Barr virus nuclear antigen 1 (EBNA1) suppresses miR-200a transcription by activating the TGFβ1-SMAD3-PI3K-AKT-c-JUN axis, thereby relieving the negative regulation of CXCL12 (12). Highly expressed CXCL12 binds to CXCR4 on the surface of regulatory T cells (Tregs), actively recruiting Tregs to the tumor site and forming a highly immunosuppressive microenvironment in NPC, as demonstrated in NPC clinical samples (13).

In addition, high-copy EBV-encoded small RNAs (EBERs) continuously induce the release of inflammatory factors such as TNF-α through pattern recognition receptors, including TLR3, establishing a state of chronic inflammation that has been observed in NPC *in vitro* systems (14).EBERs also form a positive feedback loop with LMP1 and NF-κB, further amplifying immunosuppressive signals, although this interaction has been primarily inferred from NPC model systems, and direct patient evidence remains limited.

### 2.2 Recruiting immunosuppressive cells

Under EBV influence, various immune and stromal cells are recruited or reprogrammed, collectively forming a regulatory network dominated by immunosuppressive cells, including Tregs, myeloid-derived suppressor cells (MDSCs), and Tumor-associated macrophages (TAMs) (15).

Under physiological conditions, Tregs maintain immune tolerance. In the NPC-TME, EBV enhances Treg development and suppressive activity through the CD70-CD27 interaction. Foxp3^+^ Tregs inhibit T-cell function by expressing CTLA-4 and secreting IL-10 and IL-35, which has been confirmed in NPC tissues by immunohistochemistry and flow cytometry (16). Tregs are recruited to the tumor site via chemotactic axes such as CCR4-CCL17/22 and CXCR3-CCL9/10/11; however, direct evidence for these specific axes in NPC is largely inferred from other cancer types. They suppress naive T-cell proliferation in a cell-contact-dependent manner or induce T-cell apoptosis through the perforin/granzyme B and Fas/FasL pathways, as demonstrated in NPC-infiltrating Tregs (17).

MDSCs are key mediators of the immunosuppressive TME. They highly express arginase 1 (ARG1) and inducible nitric oxide synthase (iNOS) to consume essential amino acids such as arginine in the microenvironment. MDSCs expand under glycolytic stimulation and produce reactive oxygen species (ROS) and nitric oxide (NO), disrupting T-cell metabolic homeostasis and inducing apoptosis; these functional assignments are primarily inferred from studies in other cancers, and direct evidence in NPC remains sparse (18). Furthermore, PD-L1-high MDSCs directly inhibit T-cell function via the PD-1/PD-L1 axis and secrete IL-10, TGF-β, and other factors to suppress dendritic cell (DC) maturation, blocking the initiation of T-cell activation, a mechanism partially supported by single-cell transcriptomic data from NPC patients (19).

TAMs are recruited to the tumor site under the induction of tumor-derived chemokines and cytokines, and are predominantly polarized toward an M2-like phenotype in the NPC-TME (20). M2-type TAMs highly express PD-L1, which binds to PD-1 on T cells, recruits SHP-2 to inhibit the PI3K/Akt pathway, downregulates co-stimulatory molecules, and directly suppresses T-cell activation and proliferation; this axis has been validated in NPC cell lines and patient samples (21). M2-type TAMs also secrete VEGF, IL-10, and matrix metalloproteinases (MMPs) to promote angiogenesis, degrade the extracellular matrix, and enhance tumor cell invasion, although direct evidence for these functions in NPC is largely inferred from other solid tumors (22).

However, the therapeutic efficacy of targeting these M2-associated pathways in NPC has been limited. A major reason may be that our understanding of macrophage-tumor interactions has been challenged by recent research. For instance, the classical “don’t eat me” signal CD47, a widely pursued target to block macrophage evasion, showed only a modest effect in inhibiting human macrophage phagocytosis in a genome-wide CRISPR screen; this finding has been obtained in acute myeloid leukemia (AML) models and has not yet been experimentally validated in NPC (23). Instead, the screen identified sialylated CD43 as a potent glyco-immune checkpoint that acts as a physical and electrostatic shield on the cancer cell surface, forming a “glyco-immune barrier” that restrains the cytotoxic activity of not only macrophages but also natural killer (NK) and T cells-a concept currently based on AML data and awaiting confirmation in NPC (24). This discovery suggests that therapeutic strategies targeting CD43 might be more effective than traditional CD47 blockade in reactivating anti-tumor immunity, but this remains a future direction for NPC research, as no NPC-specific evidence is yet available. Future studies are warranted to investigate CD43 expression and its functional significance in the NPC microenvironment, which could unveil a novel therapeutic vulnerability for improving ICB efficacy in this disease (25).

Notably, these cells do not act independently but form a positive feedback interactive network that stabilizes the immunosuppressive microenvironment. For example, IL-10 and TGF-β secreted by MDSCs promote Treg expansion (26); TGF-β1, CCL20, and other factors released by TAMs further recruit Tregs and inhibit DC maturation (27); and Tregs, in turn, maintain the phenotype and function of M2-type TAMs.

### 2.3 Inhibiting effector cells

Full T-cell activation requires two signals: TCR recognition of antigen (first signal) and binding of the co-stimulatory molecule CD28 to B7 molecules (CD80/CD86) on antigen-presenting cells (APCs) (second signal). CTLA-4 is structurally similar to CD28 but binds B7 with 20–50 times higher affinity, thereby physically blocking CD28-mediated co-stimulation (28). In addition, CTLA-4 binding to B7 induces trans-endocytosis to remove and degrade B7 from the APC surface, further weakening APC-dependent T-cell activation. Its intracellular domain also recruits phosphatases to negatively regulate T-cell activation pathways. These basic mechanisms have been well established in general T-cell biology and have also been observed in tumor-infiltrating Tregs from NPC patients.

Of note, Tregs highly express CTLA-4 and extensively consume B7 on APCs. This not only directly inhibits naive T-cell activation but also indirectly increases soluble PD-L1, exerting dual suppression on effector T cells, a finding confirmed in NPC Tregs (29). Furthermore, CTLs in the NPC-TME highly express inhibitory receptors such as PD-1 and TIM-3. The binding of PD-L1 on tumor cells to PD-1 directly inhibits proliferation and cytotoxicity, rendering T cells unable to effectively eliminate tumor cells; this has been directly demonstrated in NPC by single-cell sequencing and flow cytometry (30).

Beyond T-cell exhaustion, EBV-infected tumor cells downregulate MHC-I/II and natural killer (NK) cell-activating ligands (e.g., NKG2D) and secrete soluble ligands (sMICA) to competitively inhibit NKG2D activation on NK cells, drastically reducing their cytotoxicity. This immune evasion strategy has been experimentally validated in NPC cell lines and patient samples (31).

### 2.4 Physical and chemical barriers

The extracellular matrix (ECM) is a dynamic three-dimensional network composed of collagen, fibronectin, and other components that provides physical support for the TME and mediates intercellular communication. Together with cancer-associated fibroblasts (CAFs), the ECM forms a physicochemical barrier that restricts immune cell infiltration and function.

CAFs are activated by TGF-β, PDGF, and other factors and serve as key executors of this barrier (32). Activated CAFs abundantly secrete ECM components, including type I collagen and fibronectin, which are crosslinked by lysyl oxidase (LOX) to form a dense and rigid stromal network that physically impedes CD8^+^ T-cell and NK-cell infiltration into the tumor core; this physical barrier has been characterized in other solid tumors, but direct evidence in NPC remains limited (33). In addition, chemokines such as CXCL12 secreted by CAFs anchor to the ECM and form chemical gradients, trapping CXCR4-expressing T cells at the tumor periphery and creating a chemotactic exclusion barrier-a mechanism that has been validated in NPC through studies of the CXCL12-CXCR4 axis (see section 2.1) (34). CAFs also secrete VEGF, IL-6, and other factors to promote angiogenesis and further consolidate immunosuppression, which is partially supported by studies of NPC-derived CAF cultures (35).

### 2.5 Spatiotemporal heterogeneity of the TME

The NPC-TME is an immunosuppressive homeostatic system initiated by EBV, constructed via multicellular crosstalk, and capable of self-maintenance and dynamic evolution. High levels of IL-6, IL-10, TGF-β, VEGF, and other factors persist in the microenvironment. These molecules not only directly suppress effector immune cells but also continuously drive the recruitment and activation of immunosuppressive cells, forming a self-reinforcing positive feedback loop that is difficult to reverse (36). Moreover, the immunosuppressive microenvironment of NPC exhibits remarkable spatiotemporal heterogeneity, which greatly increases therapeutic complexity. Spatially, single-cell and spatial transcriptomic analyses reveal distinct immune cell compositions and functional states in the tumor core, invasive front, and lymphoid stroma (37, 38). Temporally, the microenvironment dynamically evolves with disease progression and therapeutic intervention. Such spatiotemporal heterogeneity implies that interventions targeting a single pathway or static phenotype may fail to eradicate all tumor cells, representing a major source of interpatient response heterogeneity and therapeutic resistance.

Collectively, EBV sculpts a comprehensive immunosuppressive microenvironment characterized by infiltrative immunosuppressive cells, aberrant metabolic states, fixed signaling networks, and dense physical barriers. This microenvironment not only promotes NPC initiation and progression but also directly contributes to the multiple clinical challenges of current immunotherapy. Therefore, in-depth understanding and systematic dissection of the NPC-TME represent an essential step toward developing effective combination strategies and achieving precision immunotherapy for NPC.

## 3 Mechanisms of ICB-mediated remodeling of the NPC-TME

**Figure 2:**
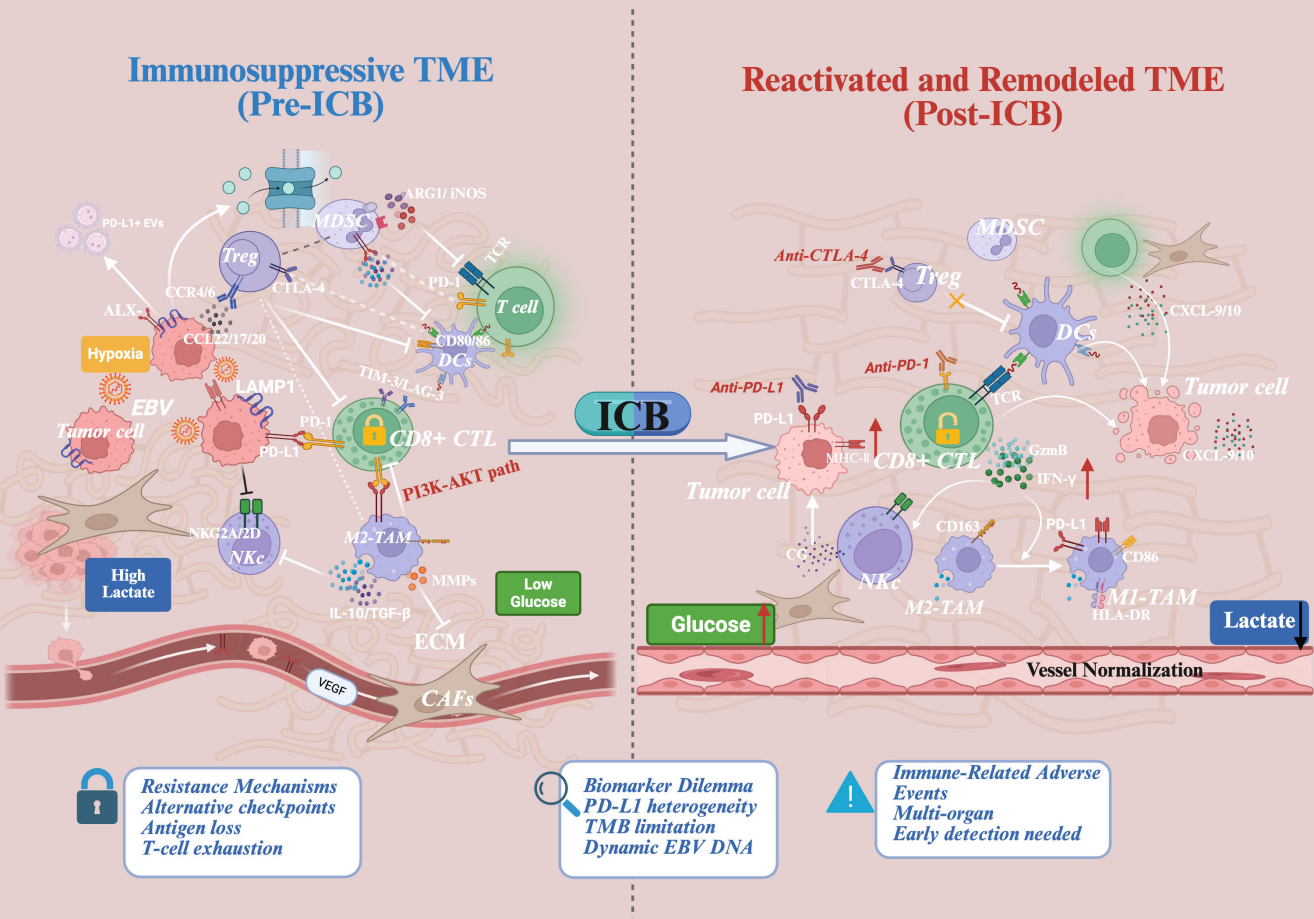
Multidimensional remodeling of the immunosuppressive NPC-TME by ICB. Left panel: EBV-infected tumor cells form a niche with nutrient deprivation (low glucose), hypoxia, and lactate accumulation, dominated by Tregs, M2-TAMs, and MDSCs that inhibit effector immunity; CD8^+^ T cells are exhausted, NK cell function is subdued, and dense extracellular matrix plus aberrant vasculature restrict immune infiltration. Right panel: Monoclonal antibodies against CTLA-4 (Tregs-expressed) and PD-L1 (tumor/myeloid cells-expressed) are administered to block these inhibitory pathways. ICB reduces immunosuppressive cells, reinvigorates CD8^+^ T/NK cells, enhances DC function, and upregulates MHC-I (increased tumor immunogenicity); the TME undergoes comprehensive normalization (restored metabolic balance, alleviated hypoxia, vessel normalization, and softened extracellular matrix barrier). Bottom panel: ICB application in NPC faces three major hurdles: diverse resistance mechanisms, biomarker dilemma, and the need for early detection/intervention of multi-organ immune-related adverse events. Image created with BioRender.com.

ICB exerts anti-NPC efficacy via gradual, coordinated TME reprogramming. Blocking PD-1/CTLA-4 relieves T-cell priming suppression, reactivating adaptive immunity to target EBV antigens. The activated immune cascade further reshapes the immunosuppressive TME by reprogramming immune cells, normalizing metabolism, and repairing vascular/physical barriers (**Figure 2**). The schematic illustrates the transition from an immunosuppressive TME (dominated by exhausted T cells and inhibitory networks) to a reactivated state following ICB intervention. It highlights interconnected mechanisms at the cellular, metabolic, and structural levels, which are dissected in detail in the following sections.

### 3.1 ICB intervenes in EBV latency and enhances antigen expression

Latent EBV infection is the predominant infection mode in undifferentiated NPC. Latent proteins encoded during EBV latency hijack antigen processing and presentation pathways, downregulate MHC-I expression on tumor cells, or directly inhibit T-cell function to achieve immune escape (39). For instance, EBV-encoded BART cluster miRNAs promote T-cell apoptosis and immunosuppression by upregulating PD-L1 in NPC cells, thereby facilitating tumor immune escape, a mechanism that has been directly validated in NPC cells (40).

ICB enhances T-cell function and increases its sensitivity to low-level EBV antigens. Meanwhile, ICB therapy induces IFN-γ secretion and activates the JAK-STAT1 pathway, thereby upregulating MHC-I expression on tumor cells, enhancing the presentation of EBV-associated antigens; this effect is primarily inferred from studies in other head and neck cancers, and direct evidence in NPC is still limited (41).

Anti-PD-1/PD-L1 monoclonal antibodies directly block the binding of PD-L1 to PD-1 on T cells, relieving LMP1-mediated suppression of T-cell function. Studies have shown that ICB disrupts ALIX, thereby reducing LMP1-induced sEV secretion, significantly decreasing PD-L1 levels in tumor cells and exosomes, and enhancing the anti-tumor immunity of CD8^+^ T cells *in vitro* and *in vivo*, findings that have been experimentally validated in NPC cell lines and xenograft models (42).

Following ICB administration, IFN-γ secreted by CD8^+^ T cells inhibits EBNA1 expression and related signaling in tumor cells. Furthermore, ICB combined with chemotherapy or targeted therapy downregulates CXCL12 production and suppresses Treg migration into tumor tissues, which has been observed in preclinical NPC models (43). Preclinical studies suggest that ICB combined with CXCR4 inhibitors further blocks Treg tumor infiltration and improves the distribution and function of effector T cells in the tumor core, providing a synergistic strategy to reverse TME immunosuppression; however, this combination has not yet been tested in NPC patients and remains a preclinical concept (44).

In summary, ICB exerts indirect and selective effects on EBV latency. By suppressing key viral proteins (LMP1, EBNA1) and enhancing viral antigen immunological visibility, ICB initiates NPC-TME remodeling and lays the foundation for immune restoration, metabolic normalization, and vascular improvement. Yet efficacy remains limited by the poor immune visibility of latently infected cells. Thus, combining ICB with EBV-targeted or antigen-boosting strategies is essential to improve treatment outcomes in EBV-associated NPC.

### 3.2 Reactivating adaptive immunity

#### 3.2.1 Reversing CTL exhaustion

Exhausted CTLs upregulate inhibitory checkpoints, including PD-1, CTLA-4, TIM-3, and LAG-3, while downregulating co-stimulatory receptors such as CD28 and 4-1BB. This exhausted phenotype has been directly observed in CTLs isolated from NPC tumors using single−cell RNA sequencing (45). PD-1 is mainly expressed on activated T cells, NK cells, and other immune subsets, whereas its ligand PD-L1 is widely distributed on tumor cells, antigen-presenting cells, and stromal cells (46).

Upon PD-1/PD-L1 ligation, the intracellular immunoreceptor tyrosine-based inhibitory motif (ITIM) and immunoreceptor tyrosine-based switch motif (ITSM) recruit tyrosine phosphatases, including SHP-1 and SHP-2, inhibiting downstream PI3K/Akt and Ras/MAPK signaling. This ultimately leads to impaired proliferation, metabolic dysregulation, and functional exhaustion of T cells. These signaling events have been well characterized in T cells across multiple cancer types, including NPC (47). ICB restores the integrity of T-cell receptor (TCR) signaling by interrupting this cascade.

Anti-PD-1 blockade disrupts PD-1/PD-L1 interaction and reinvigorates T-cell responses. It also reprograms T-cell metabolism, promotes proliferation, and enhances the expression of effector molecules, including perforin, granzymes, and cytokines, thereby reactivating cytotoxic T lymphocyte (CTL) responses (48).

Notably, ICB is unlikely to reverse exhaustion in terminally exhausted CTLs fully. Rather than directly erasing the exhaustion program, ICB relieves the suppression of stem-like CTLs and drives the proliferation of nascent functional T cells (49). However, studies have shown that reactivated CTLs exhibit limited epigenetic modifications following anti-PD-L1 therapy, and their gene expression profile reverts to an exhausted state upon treatment cessation. Whether these signaling events have been well characterized in T cells across multiple cancer types, including NPC (50).

#### 3.2.2 Relieving Treg-mediated suppression

Activated FoxP3^+^ Tregs highly express CTLA-4 and PD-L1 and induce PD-1 upregulation on effector T cells. Initially, anti-CTLA-4 antibodies were thought to antagonize inhibitory signals on activated effector T cells and restore anti-tumor activity (29). However, studies have revealed that anti-CTLA-4 monoclonal antibodies do not simply block signaling but deplete intratumoral Tregs via Fc-mediated effector functions, especially when the Fc domain is engineered for enhanced activity. This mechanism has been validated in mouse tumor models and in human cancers, though NPC-specific data remain limited (51).

In contrast to CTLA-4, the impact of PD-1 blockade on Tregs remains incompletely defined. Tregs within the TME express PD-1 at levels comparable to effector T cells, and their survival and function depend on TCR and CD28 signaling. PD-1 blockade may therefore paradoxically activate Treg immunosuppressive function, and disease hyperprogression has been documented during PD-1 blockade in some cancer types, including case reports in NPC, but systematic studies are lacking (52, 53).

Beyond these canonical pathways, recent single-cell and spatial transcriptomic analyses have uncovered an NPC-specific Treg regulatory axis involving ICOSLG-ICOS (54). In NPC, a subset of malignant cells (TUBB2B^+^) activates Tregs (TNFRSF4^+^) via the ICOSLG-ICOS pathway. These activated Tregs subsequently suppress multiple immune cell types—including T cells, NK cells, B cells, and myeloid cells—through downstream effectors such as CTLA4-CD80/86, and CD48-CD244A interactions. Importantly, the activation of this ICOSLG-ICOS network correlates with poor prognosis in NPC patients (54). Therefore, targeting this axis represents a promising strategy to relieve Treg-mediated immunosuppression in NPC. This ICOSLG-ICOS mechanism has been directly validated in primary NPC tissues by single-cell RNA sequencing.

#### 3.2.3 Synergistic blockade of PD-1/PD-L1 and CTLA-4 pathways

**Figure 3:**
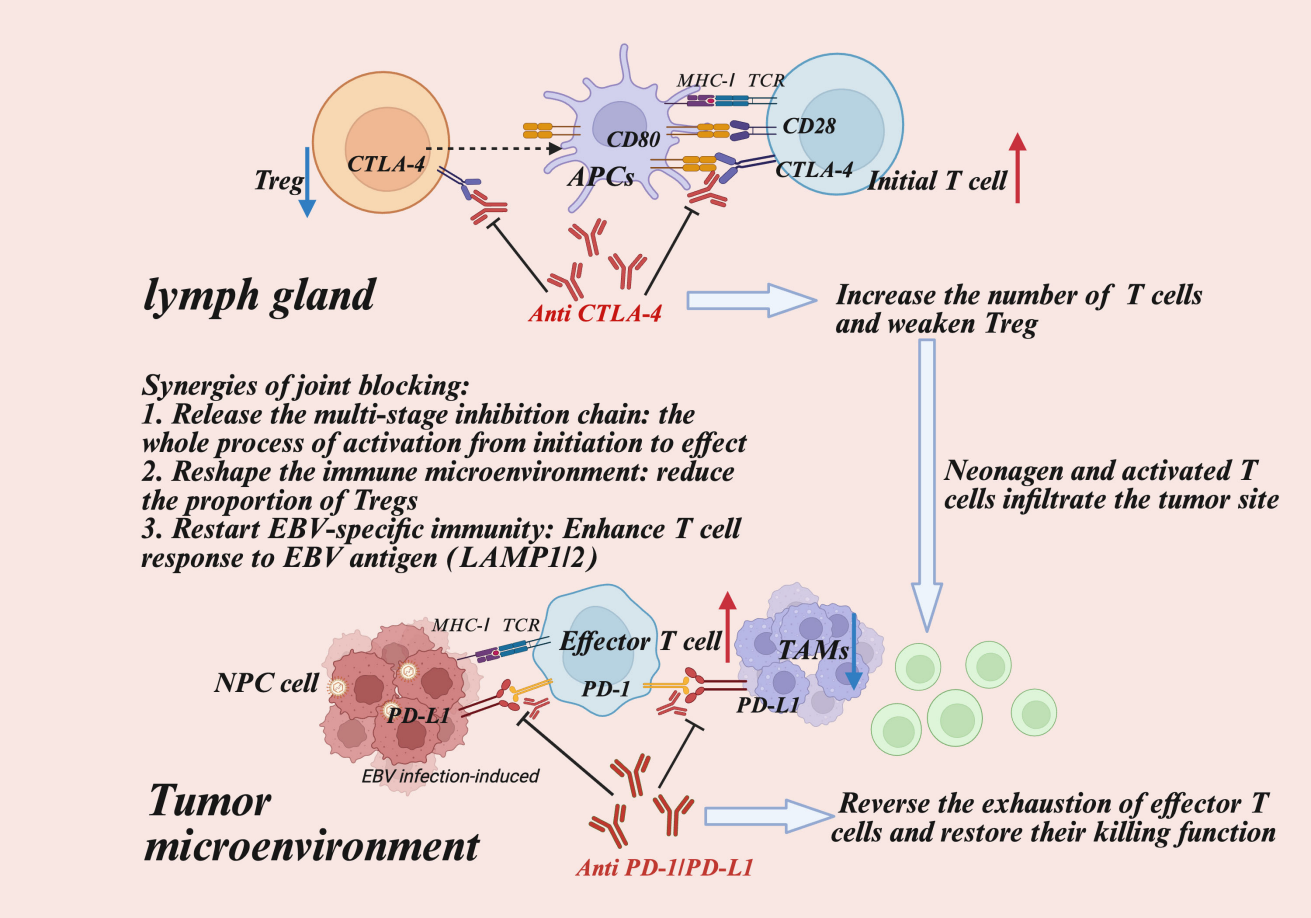
Synergistic immune activation by combined CTLA-4 and PD-1/PD-L1 blockade in NPC. The upper part illustrates the mechanism of anti-CTLA-4 blockade; the lower part illustrates the mechanism of anti-PD-1/PD-L1 blockade. The middle part shows the synergistic effects: disruption of inhibitory cascades to restore T‑cell activation and effector function; remodeling of the immune microenvironment and reduced Treg proportion; reactivation of EBV‑specific immunity and enhanced T‑cell reactivity against EBV antigens; reversal of effector T‑cell exhaustion and restored cytotoxic function. The diagram shows the targets of anti-CTLA-4 and anti-PD-1/PD-L1 antibodies and their immune regulatory effects. Image created with BioRender.com.

Relieving T-cell inhibition is central to ICB-mediated NPC-TME remodeling. The PD-1/PD-L1 and CTLA-4 pathways act synergistically and complementarily, governing the effector and priming phases of anti-tumor immunity, respectively, forming a tight immunosuppressive network (**Figure 3**).

The PD-1/PD-L1 axis dominates immune escape at the effector phase. ICB restores TCR signaling integrity by blocking PD-1/PD-L1 ligation. This not only rescues PI3K/AKT/mTOR signaling to drive T-cell proliferation and metabolic reprogramming (55), but also enables functional CD8^+^ T cells to secrete abundant IFN-γ. IFN-γ activates the JAK-STAT1 pathway, which induces MHC-I upregulation to enhance antigen presentation and promotes CXCL9/10/11 secretion to recruit more effector cells, establishing a self-amplifying anti-tumor immune cycle (56). Created with BioRender.com.

In contrast, the CTLA-4 pathway primarily suppresses T-cell priming in secondary lymphoid organs. Anti-CTLA-4 antibodies block CTLA-4/B7 interactions to unleash co-stimulation, promoting naive T-cell activation and clonal expansion (57). Together with the antigen-dependent first signal from the TCR, this activates PI3K/Akt and NF-κB, drives T-cell progression from G0 to G1 phase, and stimulates IL-2 production, ultimately leading to robust expansion and differentiation of antigen-specific T cells.

Beyond signaling blockade, Fc-mediated effector functions are critical for anti-CTLA-4 efficacy. The Fc domain induces antibody-dependent cellular cytotoxicity (ADCC) to selectively deplete intratumoral Tregs with high CTLA-4 expression, an effect enhanced by Fc engineering (58). Thus, the core role of CTLA-4 blockade is to unleash immune priming and deplete tumor-infiltrating Tregs.

Clinical studies confirm that combined CTLA-4 and PD-1 blockade yields striking synergistic anti-tumor effects. CTLA-4 inhibition promotes the generation and infiltration of tumor-specific T-cell clones, while PD-1 blockade preserves their cytotoxic function, jointly reshaping the immunosuppressive microenvironment (59). Therefore, understanding and co-targeting these two central pathways is pivotal for ICB to successfully remodel the NPC immune microenvironment and overcome resistance. The synergistic effects described here are largely derived from clinical trials in melanoma and other solid tumors; in NPC, direct evidence for such synergy is still emerging and requires further dedicated studies.

### 3.3 Reprogramming innate immunity

TAMs are abundant in the NPC-TME. PD-1/PD-L1 inhibitors induce the polarization of M2-like TAMs toward an M1-like phenotype by stimulating CD8^+^ T cells to secrete IFN-γ, significantly increasing the M1/M2 ratio, reducing M2-associated features, and enhancing phagocytic and anti-tumor functions. Beyond macrophages, ICB activates NK-cell anti-tumor function through dual mechanisms. While activating adaptive immunity, IFN-γ derived from T cells markedly upregulates activating receptors (e.g., NKG2D) on NK cells and increases intracellular stores of perforin and granzyme B, boosting cytotoxicity by 2–3-fold (60).

In cisplatin plus anti-PD-1 regimens, although chemotherapy upregulates PD-1/PD-L1, ICB specifically blocks this axis, increasing NK-cell killing of NPC cells by 40%–60% compared with chemotherapy alone, inhibiting NPC immune escape from NK cells, and reinforcing innate anti-tumor immunity (61).

### 3.4 Reversing metabolic disorders

The metabolic mechanisms summarized in **Table 1** are predominantly derived from studies in other cancer types or from basic immunology. Direct experimental validation of these specific metabolic pathways in NPC remains limited, with the exception of GLUT1 upregulation in NPC cells (62) and IDO1 expression inferred from NPC transcriptomic data.

**Table 1:** Effects of ICB in correcting metabolic dysregulation in the NPC-TME.

Metabolic dimension	TME metabolic feature	ICB intervention target	Post-intervention effect
Glucose Metabolism	Warburg effect (63) GLUT1↑ (62) Lactate↑	GLUT1/HK1/PKM2↓ GLUT3/PI3K/AKt↑	T cell function↑ DC antigen presentation↑
Lipid Metabolism	CD36↑ (64) M2 polarization (65) Fatty acid uptake↑	CD36↓ FABP4/CPT1A/AMPK↑	T cell survival time↑ M1/M2↑
Amino Acid Metabolism (66)	Tryptophan↓ Arginine↓ Kynurenine↑	IDO1/TDO-Kyn-AhR↓ ASS1↑	TCR signaling↑ Granzyme B/Perforin↑
Glutamine Metabolism	Tumor cell GLS1↑	GLS1↓ GLS2↑	Mitochondrial ATP↑ Oxidative stress damage↓

GLUT1/3, Glucose transporter 1/3; HK2, Hexokinase 2; PKM2, Pyruvate kinase M2; CD36, Fatty acid translocase; FABP4, Fatty acid binding protein 4; CPT1A, Carnitine palmitoyltransferase 1A; IDO1, Indoleamine 2, 3-dioxygenase 1; TDO, Tryptophan 2, 3-dioxygenase; Kyn, Kynurenine; AhR, Aryl hydrocarbon receptor; ASS1, Argininosuccinate synthase; GLS1/2, Glutaminase 1/2. ↑ indicates positive regulation or enhancement; ↓ indicates negative regulation or attenuation.

#### 3.4.1 Restoring glucose metabolism in immune cells

Tumor cells highly express GLUT1 and establish a glucose sink, depriving effector T cells, DCs, and other immune cells of sufficient glucose uptake. ICB restores TCR-mediated PI3K/Akt/mTOR signaling by blocking PD-1/PD-L1 and CTLA-4, directly downregulating glycolytic molecules including GLUT1, HK2, and PKM2 in NPC cells. This reduces glucose uptake and lactate extrusion, alleviates TME acidosis, and breaks the glucose sink (67).

Concurrently, ICB enhances GLUT3 and glycolytic enzyme expression in T cells, improving metabolic fitness in glucose-competitive environments and ensuring ATP and biosynthetic supply for effector function (68).

#### 3.4.2 Regulating lipid metabolism

Lipid metabolism is also severely imbalanced in the TME. Tumor cells highly express fatty acid transporters such as CD36 to avidly take up exogenous lipids, supporting rapid proliferation and stress resistance. ICB downregulates CD36 expression by modulating PI3K/Akt and AMPK signaling in tumor cells, reducing lipid scavenging (69).

Meanwhile, ICB activates AMPK and upregulates fatty acid-binding protein FABP4 and the fatty acid oxidation rate-limiting enzyme CPT1A in effector T cells, driving fatty acid oxidation as an alternative energy source under nutrient deprivation (70).

#### 3.4.3 Correcting amino acid metabolic abnormalities

EBV infection upregulates indoleamine 2,3-dioxygenase 1 (IDO1) and tryptophan 2,3-dioxygenase (TDO) in tumor and antigen-presenting cells, causing tryptophan depletion and kynurenine accumulation. Kynurenine activates the aryl hydrocarbon receptor (AhR) on T cells, inducing cell cycle arrest and mitochondrial apoptosis, inhibiting TCR and CD28 co-stimulation. Concurrent dysregulation of arginine and glutamine metabolism further exacerbates immune cell dysfunction (71, 72).

ICB targets the IDO1/TDO–Trp–Kyn–AhR inhibitory axis by suppressing STAT3, NF-κB, and other transcription factors, significantly reducing IDO1/TDO expression, limiting tryptophan catabolism, blocking kynurenine-mediated T-cell toxicity, and restoring tumor-specific recognition and killing (73).

IFN-γ from ICB-activated CD8^+^ T cells inhibits ARG1 expression in TAMs to reduce arginine degradation and upregulates argininosuccinate synthase 1 (ASS1) in T cells to regenerate arginine via the citrulline cycle, restoring TCR signaling and mTOR activation (74).

Glutamine metabolism is also rebalanced: ICB inhibits glutaminase 1 (GLS1) in tumor cells while promoting glutaminase 2 (GLS2) in T cells, prioritizing scarce glutamine for mitochondrial oxidative phosphorylation in immune cells to sustain energy and effector synthesis (75).

### 3.5 Improving the vascular and stromal microenvironment

The NPC vascular microenvironment is characterized by abnormal angiogenesis, disorganized architecture, and impaired function, leading to hypoxia, insufficient immune infiltration, and immunosuppression.

ICB inhibits HIF-1α activity via IFN-γ released by activated CD8^+^ T cells, reduces VEGF/ANGPT2 expression, promotes endothelial maturation, lowers vascular permeability, improves perfusion and oxygenation, and enhances immune cell infiltration. Meanwhile, IFN-γ activates IRF1 and drives the differentiation of high endothelial venules (HEVs) into inflammatory IFN-HEVs, which highly express CXCL9 and CXCL10 to further boost CD4^+^/CD8^+^ T-cell recruitment (76).

Single-cell analyses confirm that PD-1 blockade suppresses the endothelial CXCL12–CXCR4 axis via PD-L1 blockade, reducing tumor endothelial cell abundance and CXCL12 expression to exert anti-angiogenic effects (77). Furthermore, PD-1 downregulation inhibits HIF-1α-induced angiogenic factor expression under hypoxia, reducing immunosuppressive cell infiltration while enhancing CD8^+^ T-cell glycolysis and cytotoxicity, thereby optimizing vascular homeostasis (78). It should be noted that most of the vascular and stromal remodeling mechanisms described in this section have been characterized in other solid tumors; direct experimental evidence in NPC is still sparse, and many of these findings should be considered inferred from other cancer types.

ICB indirectly transforms the ECM from an inhibitory physical barrier into a permissive scaffold for immune infiltration and function by modulating ECM-producing, regulatory, and degrading cells.

IFN-γ secreted by activated CTLs inhibits CAF secretion of pro-fibrotic factors (TGF-β, PDGF) and ECM components (collagen I, fibronectin) (79). Suppressed CAFs produce less LOX and other cross-linking enzymes, reducing collagen cross-linking and ECM stiffness, thereby limiting the deposition of dense, rigid matrix (80).

ICB-mediated CAF inhibition and ECM remodeling also disrupt the CXCL12-dependent chemical barrier, enabling effective T-cell penetration into the tumor parenchyma (81). ICB-induced immune activation shifts the balance between matrix metalloproteinases (MMPs, ECM-degrading) and tissue inhibitors of metalloproteinases (TIMPs, ECM-protective), promoting macrophage MMP expression and modulating the MMP/TIMP profile across multiple cell types (82).

### 3.6 Peripheral nerve–tumor crosstalk

The head and neck region is densely innervated by peripheral nerves, raising the possibility of bidirectional interactions between neural components and NPC cells, a concept that has gained substantial traction in the broader field of cancer neuroscience but remains largely unexplored in NPC (83). In other cancer types, at least two major modes of nerve–tumor interaction have been recognized: perineural invasion (PNI), where cancer cells infiltrate the adjacent nerve sheaths, and tumor innervation, where axons actively extend into tumor bodies (84). Beyond their direct physical interplay, emerging evidence has positioned neural elements as an integral part of the TME, forming a “neuro-immune axis” that profoundly influences tumor biology (85).

On one hand, tumor cells actively secrete neurotrophic factors (such as nerve growth factor (NGF) and brain-derived neurotrophic factor (BDNF)) and chemotactic signals to promote nerve recruitment, axonogenesis, and neural reprogramming (83, 86). Reciprocally, activated peripheral nerves—particularly sympathetic and sensory fibers—release neurotransmitters (e.g., norepinephrine, substance P) and neuropeptides that can directly modulate tumor cell behavior or, importantly, reshape the immune landscape of the TME (87). For instance, adrenergic signaling has been shown to suppress dendritic cell priming, impair natural killer (NK) and CD8^+^ T cell cytotoxicity, and polarize myeloid cells toward an immunosuppressive phenotype, collectively promoting immune evasion and tumor progression (88).

Despite growing appreciation of the nerve–tumor crosstalk in various malignancies, direct evidence in NPC remains critically limited. Whether peripheral nerves infiltrate NPC tissues, whether specific neurotransmitter pathways are active in the NPC-TME, and whether such interactions influence response to ICB are all open questions. Given that the cranial nerve-adjacent anatomical location and frequent cranial nerve involvement in advanced NPC, investigating the neuro-immune axis in this disease may unveil novel therapeutic vulnerabilities and provide a rationale for combining neuromodulatory agents (e.g., β-blockers targeting adrenergic signaling) with existing immunotherapies (86). Future studies integrating spatially resolved transcriptomics, clinical NPC specimens, and preclinical models are urgently needed to decipher these underexplored nerve–tumor interactions in NPC.

## 4 Clinical efficacy and challenges of ICB in NPC

### 4.1 Clinical trial progress and efficacy data

**Table 2** summarizes efficacy and safety data from key ICB clinical trials in recurrent/metastatic and locally advanced NPC, highlighting its clinical value and stage-specific features to support mechanistic analyses and rational combination strategies.

**Table 2:** Summary of efficacy and safety data from key ICB clinical trials in nasopharyngeal carcinoma.

Diseases stage	Trial ID	Phase	Treatment regimen	Median follow-up	Sample size (n)	Primary efficacy endpoint (vs. control)	Safety data (≥3 TRAEs)	Clinical significance
R/M NPC	(NCT03581786) (89)	III	Toripalimab + GP vs Placebo + GP	36 months	289	mPFS: 21.4 vs 8.2 mo (HR 0.52); mOS: 36.0 vs 29.7 mo (HR 0.60)	89.7% vs 90.3%	Established ICB+GP as standard first-line therapy for R/M NPC in China
R/M NPC	(NCT03707509) (90)	III	Camrelizumab + GP vs Placebo + GP	NA	263	mPFS: 10.8 vs 6 months (HR 0.51)	93% vs 90%	NMPA approval for first-line treatment of recurrent/metastatic nasopharyngeal carcinoma
R/M NPC	(NCT03924986) (91)	III	Tislelizumab + GP vs Placebo + GP	NA	263	mPFS: 9.6 vs 7.4 mo (HR 0.50); 2y OS rate: 74.6% vs 62.6%	88.1% vs 89.3%	NMPA approved for first-line treatment of R/M NPC
R/M NPC	(NCT02915432) (92)	II	Toripalimab (monotherapy)	NA	190	ORR: 20.5%mPFS: 1.9 months mOS: 17.4- -month	12.6%	First study demonstrating durable efficacy of PD-1 monotherapy
R/M NPC	(NCT02611960) (93)	III	Pembrolizumab vs Chemotherapy	NA	228	mOS: 17.2 vs 15.3 mo (HR 0.90, p=0.226)	14% vs 35%	Showed survival trend and better safety for PD-1 monotherapy
LANPC	(NCT03700476) (94)	III	Sintilimab + Standard CRT vs Standard CRT	41.9 months	425	3y EFS: 86.1% vs. 76.0% (HR 0.59)	74% vs 65%	Chemoradiotherapy + Sintilimab improved EFS
LANPC	(NCT03984357) (95)	II	Nivolumab + Chemoradiotherapy	43 months	152	3y FFS: 88.5%; 3y OS: 97.9%	5.2%	Demonstrated favorable anti-tumor activity and low toxicity
LANPC	(NCT03925090) (96)	II	Toripalimab + CCRT + Toripalimab vs Placebo + CCRT + Placebo	37.5 months	150	2y PFS: 92% vs 74% (HR 0.29)	40.0% vs 44.4%	Toripalimab combined with concurrent chemoradiotherapy is promising
LANPC	(NCT03427827) (97)	III	Radical CRT + Adjuvant Camrelizumab	39 months	450	3y EFS: 86.9% vs. 77.3% (HR 0.56; P = 0.01)	11.2% vs 3.2%	Adjuvant PD-1 blockade with camrelizumab significantly improved EFS with manageable toxicity.

ICB, immune checkpoint inhibitor; R/M NPC, recurrent/metastatic nasopharyngeal carcinoma; LANPC, locally advanced nasopharyngeal carcinoma; GP, gemcitabine + cisplatin; mPFS, median progression-free survival; mOS, median overall survival; HR, hazard ratio; ORR, objective response rate; TRAEs, treatment-related adverse events; NMPA, National Medical Products Administration; CCRT, concurrent chemoradiotherapy.

Explanations: 1) Efficacy based on intention-to-treat population; 2)HR < 1.0 favors ICB; 3)TRAEs: grade ≥3; 4)NA, not available/immature. Trials completed enrollment and reported key data as of February 2025. Regulatory status varies by region.

### 4.2 EBV-driven specific resistance

Although ICB combined with chemotherapy has significantly improved survival outcomes in R/M NPC, resistance remains the core bottleneck limiting its clinical benefit. Similar to other solid tumors, NPC resistance can be attributed to both tumor cell-intrinsic adaptation and adaptive remodeling of the tumor microenvironment (98).

Tumor cell-intrinsic loss of immunogenicity forms the foundation of resistance, a process largely actively orchestrated by EBV in NPC. LMP1-mediated NF-κB upregulates DNA methyltransferase 3b (DNMT3b) expression, inducing increased methylation of the PTEN CpG island, silencing the major tumor suppressor PTEN, and driving immune escape-a mechanism that has been validated in NPC cell lines. Additionally, LMP1 upregulates PD-L1 expression by promoting the interaction between protein arginine methyltransferase 1 (PRMT1) and PGC-1α, actively reducing viral antigen density to evade CTL recognition (99); this has been directly demonstrated in NPC cells. Single-cell sequencing has confirmed that tumor subclones in this antigen-silenced state undergo positive selection under anti-PD-1 therapy pressure, providing direct evidence from NPC patients (100).

ICB may induce compensatory expression of immune checkpoints such as LAG-3, TIM-3, and TIGIT. Combined with the positive feedback crosstalk of the Treg-M2 TAM-MDSC axis and the irreversibility of exhaustion-related epigenetic programs in T cells, these factors collectively drive acquired resistance (101).In NPC, the depth of remodeling and spatial organization of this network differ significantly from those of other solid tumors. Single-cell analysis reveals a highly enriched subpopulation of CD8^+^ T cells with triple exhaustion (PD-1^+^/TIM-3^+^/LAG-3^+^) in resistant lesions, which are completely unresponsive to single PD-1 blockade (102). Spatial transcriptomics further demonstrates that Tregs, M2-type TAMs, and MDSCs exhibit immunosuppressive fortress-like co-localization and enrichment in resistant NPC, with spatial density far exceeding that in lung cancer and melanoma (103). Critically, EBV-encoded BART cluster miRNAs can be directly transferred to TAMs and Tregs via exosomes, inducing M2 polarization and stabilizing FOXP3 expression, respectively; these findings are based on NPC exosome studies (104).

Active adaptation of the EBV latency program represents the most characteristic resistance mechanism in NPC. This active adaptation manifests in three identifiable patterns: first, adaptive downregulation of antigen expression, where tumor cells silence LMP1 via promoter methylation or inhibit LMP2/EBNA1 synthesis through BART cluster miRNA-mediated translational repression, a pattern confirmed in NPC specimens (105); second, the double-edged sword effect of the latent-lytic switch, where low-level expression of lytic genes (BZLF1, BALF5) occurs in some resistant lesions. While lytic activation transiently releases viral antigens, the lytic protein vIL-10 homolog BCRF1 exerts direct immunosuppressive functions. Moreover, lytic-induced pyroptosis may recruit MDSCs by releasing IL-1β and ATP, such that the net effect remains skewed toward immune escape (106); third, remote regulation of the TME by EBV-miRNAs (as noted earlier), in which exosome-carried viral miRNAs can reprogram host immune cell phenotypes across cell types, with inhibitory depth and duration far exceeding those of soluble cytokines (107).

### 4.3 Limitations and emerging directions of biomarkers

Currently, there is a lack of reliable biomarkers for accurately identifying ICB-benefiting patient populations, limiting the precise application of therapy.

PD-L1 is the most commonly used biomarker, but its predictive value is limited in NPC. NPC typically exhibits low-to-moderate tumor mutational burden (TMB). Plasma EBV DNA is a unique prognostic biomarker for NPC, but studies on its correlation with ICB efficacy yield inconsistent conclusions, precluding its use as a standalone decision-making tool and requiring integration with other immune indicators for interpretation (108). The NPC-TME is rich in immune cell infiltration; however, as their functional status is regulated by multiple inhibitory cells and signals, simple assessment of CD8^+^ T-cell density is insufficient to predict efficacy (109). Non-invasive biomarkers such as circulating tumor DNA (ctDNA) show potential for dynamic monitoring of clonal evolution, but their sensitivity and specificity for predicting ICB efficacy in NPC require validation in large-scale prospective studies (110).

Beyond these conventional markers, several emerging biomarker classes have garnered increasing attention and warrant further exploration in NPC.

First, immune gene signatures derived from bulk or single-cell transcriptomics offer a composite view of the tumor immune microenvironment. For example, an IFN-γ-related T cell-inflamed signature-encompassing genes such as CXCL9, CXCL10, and CD274 (PD-L1) have been associated with response to ICB across multiple cancer types (111). Whether similar signatures predict ICB outcomes in NPC requires prospective validation.

Second, T-cell functional states provide a more dynamic perspective than simple cell density measurements. The co-expression of exhaustion markers (e.g., TIM-3, LAG-3, TIGIT) along with activation markers can delineate dysfunctional T-cell subsets that may fail to respond to PD-1/PD-L1 blockade (112). Multiparameter flow cytometry or imaging mass cytometry of NPC biopsies could reveal whether specific exhaustion patterns correlate with primary resistance to ICB.

Third, spatial immune architecture adds a critical topographic dimension. The physical distribution of immune cells—for instance, CD8^+^ T cells localized at the invasive margin versus those excluded from the tumor core—can influence ICB efficacy. Using digital spatial profiling (DSP), studies in NPC have demonstrated significant spatial heterogeneity of immune cell populations within the tumor microenvironment (113). Moreover, integration of spatial transcriptomics and single-cell analyses led to the development of a CTRscore (CXCL9-TLS response-predictive scoring system), which robustly forecasts ICB outcomes in independent NPC cohorts (114). Studies in other head and neck cancers have shown that immune-excluded or immune-desert phenotypes are associated with poor response. Multiplex immunofluorescence (mIF) and DSP technologies are now available to dissect such spatial heterogeneity in NPC tissues, although systematic studies are still lacking.

Collectively, no single biomarker is likely to suffice given the complexity of the NPC-TME. The absence of multi-biomarker integration models limits predictive accuracy, as single indicators cannot capture individual heterogeneity. Furthermore, difficulties in obtaining post-treatment tumor tissue, the immature state of liquid biopsy biomarkers, and disparities in detection technology accessibility hinder clinical translation. Future efforts should prioritize integrated models combining clinical, genomic, transcriptomic, and spatial parameters to precisely identify ICB-benefiting populations in NPC.

### 4.4 Immune-related adverse events

By relieving immune suppression, ICB may also cause excessive immune system activation, leading to attacks on normal tissues and resulting in immune-related adverse events (irAEs). irAEs can involve multiple organs, including the skin, gastrointestinal tract, endocrine system, liver, and lungs (115).

Cutaneous irAEs (irCAEs) have the highest incidence, mainly presenting as pruritus, erythematous rashes, and dome-shaped papules (116). Incidence varies significantly across ICB types: anti-CTLA-4 agents (e.g., ipilimumab) cause maculopapular rashes in up to 60% of patients, significantly higher than anti-PD-1/PD-L1 agents (24%) (117). Combination therapy further increases risk, with irCAE incidence (59%–72%) higher than monotherapy (118). Gastrointestinal irAEs have diarrhea and colitis as the main manifestations (119). The risk of moderate-to-severe colitis with anti-CTLA-4 agents is significantly higher than with PD-1 inhibitors, and combination therapy further elevates this risk (120). For hepatotoxicity, the incidence is 2%–10% at 6–12 weeks after monotherapy, while combination therapy results in grade ≥3 hepatitis in 25%–30% of patients (121). Endocrine toxicity has an overall incidence of approximately 10%, leading to thyroid dysfunction, hypophysitis, and type 1 diabetes (122). A retrospective cohort study showed that 42% of ICB-treated patients developed thyroid-related irAEs, with subclinical thyrotoxicosis being the most common (123). Hypophysitis incidence varies by agent: 6.4% with nivolumab plus ipilimumab versus only 0.1% with atezolizumab (124). ICB-related diabetes typically onset shortly after the first dose and is associated with a higher incidence of ketoacidosis (125). Although rare, myocarditis, pneumonitis, myasthenia gravis, and encephalitis can be life-threatening, posing significant diagnostic and management challenges that require high vigilance and multidisciplinary collaboration.

To overcome the clinical challenges of ICB in NPC, a mechanistic understanding of the root causes of resistance and toxicity is required, along with the establishment of early recognition and standardized management protocols for irAEs.

## 5 Conclusions

The immunosuppressive TME in NPC is not a passive consequence, but an actively constructed and dynamically evolving immune-escape ecosystem shaped by EBV through latent proteins and non-coding RNAs. Although ICB can reverse T-cell exhaustion, reprogram macrophage polarization, normalize metabolic imbalance, and alleviate vascular stromal barriers, its clinical efficacy is persistently limited by EBV-driven antigen silencing, compensatory TME remodeling, and irreversible epigenetic programs of T-cell exhaustion. The limited performance of current predictive biomarkers in NPC largely reflects the inadequacy of static, single-dimensional detection paradigms in the face of EBV-associated spatiotemporal heterogeneity. Future breakthroughs will depend not merely on ICB administration, but on repositioning EBV from an etiological factor to a therapeutic target, and the TME from a protective barrier to an actionable pathway.

Based on the current development stage, emerging strategies can be categorized as follows.

### 5.1 Clinically tested strategies

A combination of ICB with anti-angiogenic agents (e.g., apatinib, lenvatinib) is being evaluated in phase II/III studies. Circulating EBV DNA is already used as a real-time, non-invasive biomarker for response monitoring. These clinically tested regimens provide immediate opportunities to improve patient stratification and combination therapy.

### 5.2 Preclinical approaches

Direct targeting of EBV latency using small-molecule inhibitors, PROTAC degraders against LMP1/EBNA1, and mRNA therapeutic vaccines has shown efficacy in NPC models. Combination with epigenetic modifiers (e.g., EZH2 inhibitors, DNA demethylating agents) can reverse T-cell exhaustion and reactivate cryptic EBV antigens. Targeting key metabolic nodes such as IDO1, CD36, and ARG1 can overcome metabolic barriers, including tryptophan depletion, lipid peroxidation, and arginine deprivation.

### 5.3 Future perspectives

Intelligent delivery systems adapted to nasopharyngeal anatomy, such as TME-responsive nanocarriers, engineered tumor-associated macrophage “living drug factories”, and intraluminal platforms, represent conceptual approaches for precise spatiotemporal intervention. Combining single-cell multi-omics and spatial transcriptomics to establish a comprehensive spatiotemporal map of EBV dynamics, and using machine learning to integrate radiomics, metabolomics, and spatial TME transcriptomics, could eventually yield predictive models for resistance evolution and irAE risk. This research framework, anchored in viral biology, guided by spatiotemporal TME mapping, and centered on mechanism−based synergy, is poised to transform NPC from a prototypic EBV−associated malignancy into a paradigm disease for precision cancer immunotherapy.
